# Trends in Out-of-Pocket Healthcare Expenses Before and After Passage of the Patient Protection and Affordable Care Act

**DOI:** 10.1001/jamanetworkopen.2021.5499

**Published:** 2021-04-09

**Authors:** Krishna Vangipuram Suresh, Kevin Wang, Adam Margalit, Amit Jain

**Affiliations:** 1 Department of Orthopaedic Surgery, Johns Hopkins University School of Medicine, Baltimore, Maryland

## Abstract

This cross-sectional study analyzes trends in out-of-pocket (OOP) health expenses in the United States during the last 2 decades and compares the distribution of services that contribute most to OOP spending.

## Introduction

One goal of the 2010 Patient Protection and Affordable Care Act (ACA) was to limit patient out-of-pocket (OOP) health expenses. This cross-sectional study aimed to analyze trends in OOP health expenses in the United States during the last 2 decades and compare the distribution of services that most contribute to OOP spending.

## Methods

This study followed the Strengthening the Reporting of Observational Studies in Epidemiology (STROBE) reporting guideline for cross-sectional studies. Institutional review board approval and informed consent were waived because the data were publicly available. Data from the National Health Expenditures (NHE) Accounts from 2000 to 2018 were analyzed. OOP expenditures represent total payments made in the form of deductibles, coinsurance, and health and flexible savings accounts and by individuals who are uninsured. Expenditure estimates were converted to per capita estimates, and the Consumer Price Index was used to adjust all values to 2018 US dollars. Significance was determined using linear regression with gamma error distribution and log link. Significant changes in trends between pre-ACA (2000-2009) and post-ACA (2010-2018) OOP health expenses were determined using an interaction term between ACA and calendar year within the regression. Statistical analyses were 2-tailed (α < .05) and performed using Stata version 15.0 (StataCorp).

## Results

From 2000 to 2018, total OOP per capita health expenses increased from $1028 to $1148. The average annual growth rate (AAGR) of OOP spending significantly decreased following the ACA (mean [SD], 0.2% [1.1%] vs 1.0% [2.3%]; linear regression *P* = .001; quadratic *P* = .001) (Figure). Mean (SD) AAGR for OOP spending increased for physician services from pre-ACA to post-ACA periods (0.5% [2.1] to 0.8% [2.2]) but decreased for other components of health care cost (Table). Total per capita health expenditures increased from $6649 to $10 627 from 2000 to 2018, with a pre-ACA AAGR (SD) of 3.4% (2.2%) and post-ACA AAGR (SD) of 1.9% (1.6%) (*P* for trend < .001).

**Figure.**
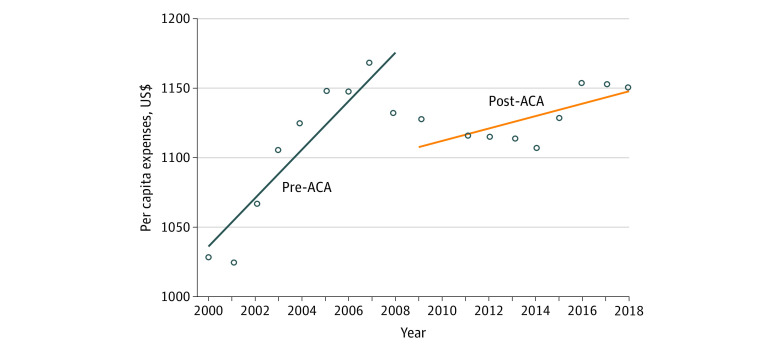
Changes in Out-of-Pocket Expenses Before and After the Patient Protection and Affordable Care Act (ACA) Dots represent the mean per capita OOP expenses for total health care expenditures at a given year. Lines of best fit are included to demonstrate overall trends over time.

**Table.** Summary of OOP Spending Trends in Components of Total Health Care Expenses

Expense	OOP spending, mean, $			AAGR, % (SD)		*P* value^a^
	2000	2009	2018	2000-2009	2010-2018	
Services						
Physician	164.78	173.25	187.00	0.5 (2.1)	0.8 (2.2)	.006
Dental	141.44	163.89	168.00	1.7 (3.4)	0.3 (2.4)	.03
Medications						
Nonprescription	156.03	182.62	196.00	1.7 (2.4)	0.8 (1.8)	<.001
Prescription	173.52	189.64	144.00	1.1 (5.8)	−2.9 (3.5)	<.001
Nonphysician and/or nondental services	56.87	66.73	80.00	1.8 (1.7)	2.0 (1.7)	<.001

Abbreviations: AAGR, average annual growth rate; OOP, out-of-pocket.

^a^ Changes in trends in OOP health expenses before and after the Patient Protection and Affordable Care Act.

## Discussion

Compared with the pre-ACA period, OOP spending increased at a slower rate for almost all health care services during the post-ACA period. Tax legislation in 2003 encouraged employers to provide high deductible health plans (HDHPs) to their employees; however, HDHPs do not reduce OOP spending because of the high upfront cost of care.^1^ The ACA implemented OOP spending maximums and increased access to preventive care services, which may have counteracted high OOP spending in HDHP plans. However, ACA-mandated price ceilings are still significant—$7900 in 2019—which may explain why OOP spending is still increasing annually.^2^ We speculate that ACA-imposed spending limits for HDHPs account for substantial OOP savings. Furthermore, access to coverage for individuals who were previously uninsured may account for additional OOP savings.

AAGRs for OOP health expenses have increased for physician services since the introduction of the ACA, possibly because of increased use of out-of-network care. Cooper et al^3^ demonstrated that at in-network hospitals, some services provided by anesthesiologists, pathologists, or assistant surgeons were billed as out-of-network services, with patients held responsible for additional costs. These costs may come under control with the passage of recent federal legislation to limit surprise billing.

OOP health expenses for prescription medications decreased rapidly from 2010 to 2018. Reasons for these findings include increased prevalence of prescription to nonprescription switches for medications, increased number of clinicians using nonprescription medications as first-line management, loss of patent protection for name-brand drugs, and increased use of prior authorization for prescriptions.^1,4,5,6^

Although the ACA may provide a partial explanation for OOP spending trends, we cannot definitively attribute these changes to the ACA alone. Coinciding economic events, such as the Great Recession, likely decreased consumer willingness to pay OOP for health-associated costs. In the current study, we were unable to determine whether these decreases were secondary to disproportionately high rates of Baby Boomers becoming eligible for Medicare. Moreover, savings in OOP health spending may be nullified by increased taxpayer spending on Medicaid. Access to coverage for individuals who were previously uninsured through Medicaid expansion may account for the greatest OOP savings.
